# The antidepressant drug sertraline is a novel inhibitor of yeast Pah1 and human lipin 1 phosphatidic acid phosphatases

**DOI:** 10.1016/j.jlr.2024.100711

**Published:** 2024-11-20

**Authors:** Geordan J. Stukey, Matthew R. Breuer, Natalie Burchat, Ruta Jog, Kollin Schultz, Gil-Soo Han, Matthew S. Sachs, Harini Sampath, Ronen Marmorstein, George M. Carman

**Affiliations:** 1 Department of Food Science, Rutgers University, New Brunswick, NJ, USA; 2 Rutgers Center for Lipid Research, Rutgers University, New Brunswick, NJ, USA; 3 Department of Biology, Texas A&M University, College Station, TX, USA; 4 Department of Nutritional Sciences, Rutgers University, New Brunswick, NJ, USA; 5 Abramson Family Cancer Research Institute, Perelman School of Medicine, University of Pennsylvania, Philadelphia, PA, USA; 6 Graduate Group in Biochemistry & Molecular Biophysics, Perelman School of Medicine, University of Pennsylvania, Philadelphia, PA, USA; 7 Department of Biochemistry & Biophysics, Perelman School of Medicine, University of Pennsylvania, Philadelphia, PA, USA

**Keywords:** phosphatidic acid phosphatase, Pah1, lipin 1, diacylglycerol, triacylglycerol, sertraline, propranolol

## Abstract

Phosphatidic acid phosphatase (PAP) is an evolutionarily conserved eukaryotic enzyme that catalyzes the Mg^2+^-dependent dephosphorylation of phosphatidic acid to produce diacylglycerol. The product and substrate of PAP are key intermediates in the synthesis of triacylglycerol and membrane phospholipids. PAP activity is associated with lipid-based cellular defects indicating the enzyme is an important target for regulation. We identified that the antidepressant sertraline is a novel inhibitor of PAP. Using *Saccharomyces cerevisiae* Pah1 as a model PAP, sertraline inhibited the activity by a noncompetitive mechanism. Sertraline also inhibited the PAP activity of human lipin 1 (α, β, and γ), an orthologue of Pah1. The inhibitor constants of sertraline for the *S. cerevisiae* and human PAP enzymes were 7-fold and ∼2-fold, respectively, lower than those of propranolol, a commonly used PAP inhibitor. Consistent with the inhibitory mechanism of sertraline and propranolol, molecular docking of the inhibitors predicts that they interact with non-catalytic residues in the haloacid dehalogenase-like catalytic domain of Pah1. The Pah1-CC (catalytic core) variant, which lacks regulatory sequences, was inhibited by both drugs in accordance with molecular docking data. That Pah1 is a physiological target of sertraline in *S. cerevisiae* is supported by the observations that the overexpression of *PAH1* rescued the sertraline-mediated inhibition of *pah1*Δ mutant cell growth, the lethal effect of overexpressing Pah1-CC was rescued by sertraline supplementation, and that a sublethal dose of the drug resulted in a 2-fold decrease in TAG content.

Phosphatidic acid (PA) phosphatase (PAP, 3-*sn*-phosphatidate phosphohydrolase, EC 3.1.3.4) is an evolutionarily conserved eukaryotic enzyme (1, 2, 3, 4, 5, 6, 7, 8, 9) that catalyzes the Mg^2+^-dependent dephosphorylation of PA to produce diacylglycerol (DAG) (10, 11) (Fig. 1A). PAP activity from diverse eukaryotic organisms is dependent on the D*X*D*X*(T/V) catalytic motif in the haloacid dehalogenase (HAD)-like the domain of the enzyme (2, 12, 13). PAP is an extensively phosphorylated enzyme, and the posttranslational modification regulates enzyme activity, subcellular localization, and protein stability (14, 15, 16). The enzyme plays a major role in lipid homeostasis by controlling the cellular levels of PA and DAG, which are key intermediates for the synthesis of triacylglycerol (TAG) and membrane phospholipids (15, 16, 17). PA and DAG also function in lipid signaling pathways (15, 18, 19, 20, 21, 22, 23), vesicular trafficking (24, 25, 26, 27, 28), lipid droplet formation (29, 30), phospholipid synthesis gene expression (31, 32, 33), and facilitate membrane fission/fusion events (34, 35, 36, 37, 38, 39). The importance of PAP to lipid homeostasis and cell physiology is exemplified in yeast, mice, and humans by a host of cellular defects (e.g., aberrant nuclear membrane morphology, defects in lipid droplet formation, fatty acid-induced lipotoxicity, defects in vacuole fusion and autophagy, apoptosis, and reduced chronological life span) and lipid-based diseases (e.g., lipodystrophy, obesity, inflammation, insulin resistance, peripheral neuropathy, type 2 diabetes) that are associated with loss or overexpression of the enzyme (15, 17, 40, 41, 42, 43, 44, 45, 46, 47, 48, 49).

**Fig. 1:**
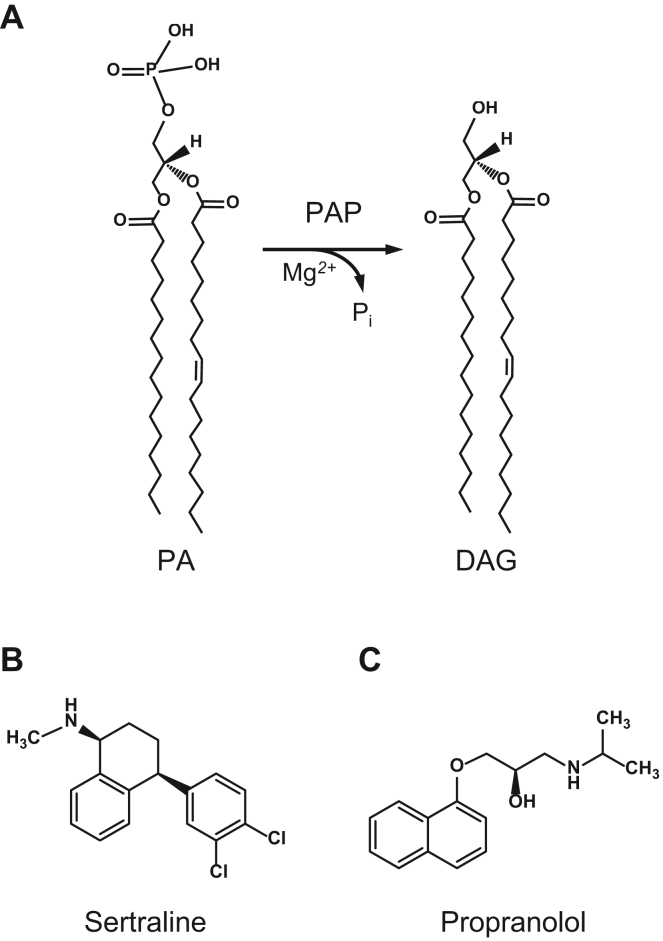
PAP reaction and structures of sertraline and propranolol. A: PAP catalyzes the Mg^2+^-dependent dephosphorylation of PA to produce DAG. The structures of PA and DAG are shown with 16:0 and 18:1 acyl chains (B), the structure of sertraline. C: the structure of propranolol.

Because of its key role in lipid synthesis, the PAP enzyme can be considered a drug target to alleviate metabolic disorders associated with the disturbance of the PA/DAG balance (17). Yet molecules that effectively regulate PAP activity are relatively rare. One molecule that is widely used to inhibit PAP activity in eukaryotic cells is propranolol (Fig. 1C) (4, 50, 51, 52, 53, 54, 55, 56, 57, 58, 59, 60). In some studies, the use of propranolol has provided insight into the role PAP activity has in cellular processes (28, 59, 60, 61, 62, 63). For example, its inhibitory effect on the PAP activity in the rice blast fungus *Magnaporthe oryzae* is the basis for alterations in lipid metabolism and suppression of fungal sexual reproduction, sporulation, growth, and infection of diverse plants (55).

Sertraline is a selective serotonin reuptake inhibitor prescribed to humans for the treatment of depression and social anxiety disorders (64). Interestingly, sertraline also exhibits antifungal activity (65, 66, 67, 68, 69, 70), but the basis of its antifungal action is unclear (65, 71). Studies with baker’s yeast *Saccharomyces cerevisiae* have led to the hypothesis that sertraline-induced lethality is a consequence of phospholipidosis, a condition that is associated with abnormal internal membrane structures (71, 72). In addition, the drug is known to induce the formation of supersized lipid droplets in *S. cerevisiae*, as well as in pathogenic yeasts *Cryptococcus neoformans* and *Candida albicans*, and the pathogenic filamentous fungus *Aspergillus fumigatus* (65). The aberrant expansion of the nuclear/endoplasmic reticulum membrane and defects in lipid droplet formation are phenotypes of PAP-deficient *S. cerevisiae pah1*Δ cells (12, 30, 40, 73, 74). Moreover, a recent study has shown that sertraline downregulates adipogenic pathways and upregulates phospholipid synthesis in human mesenchymal stem cells (75), characteristics observed in higher eukaryotes that lack lipin 1 PAP activity (42). In this work, we demonstrate that sertraline (Fig. 1B) is a novel noncompetitive inhibitor of PAP, and its potency is superior to propranolol, expanding the toolbox of reagents that can be used to control the enzyme activity in eukaryotic organisms.

## Materials and Methods

### Materials

All chemicals were reagent grade. Standard media for the growth of yeast and bacteria were acquired from Difco Laboratories. IgG-Sepharose and Q-Sepharose were purchased from GE Healthcare. Nickel-nitrilotriacetic acid agarose resin and kits for plasmid purification were from Qiagen. Pierce Strong Anion Exchange Mini Spin columns, Dulbecco’s Modified Eagle Medium, and BODIPY 493/503 were purchased from Thermo Fisher Scientific. Invitrogen-supplied DNA size ladders. Alkaline phosphatase, ampicillin, ATP, bovine serum albumin, nucleotides, Roswell Park Memorial Institute (RPMI)-1640 medium (product number R1383), silica gel 60 TLC plates, Triton X-100, and propranolol (product number P 5544) were from Millipore-Sigma. Sertraline (product number 047897) was purchased from Matrix Scientific. Roche was the source for EDTA-free cOmplete ULTRA protease inhibitor tablets. Fisher Bioreagents provided the chloramphenicol. Lipids were acquired from Avanti Polar Lipids. Radiochemicals were purchased from Revvity. National Diagnostics was the source of scintillation counting supplies.

### Cells, plasmids, and growth conditions

The cells and plasmids used in this study are listed in Table 1. Standard methods were used to culture *S. cerevisiae* and *E. coli* cells (76, 84). The growth of yeast and bacterial cells was spectrophotometrically monitored by measuring the absorbance at 600 nm (A_600 nm_). Solid growth media contained 2% and 1.5% agar for yeast and *E. coli*, respectively. Plasmid propagation was performed with *E. coli* strain DH5α. *E. coli* cells were grown at 37°C in lysogeny broth medium (1% tryptone, 0.5% yeast extract, 1% NaCl, pH 7.0). The indicated antibiotics were used to select for *E. coli* cells containing plasmids. Yeast transformants were grown in synthetic complete (SC) medium-2% glucose lacking the appropriate nutrient for plasmid maintenance.

**Table 1:** Cells and plasmids used in this study

Cell or plasmid	Genotype or Relevant Characteristics	Source or Reference
Cell		
*Escherichia coli*		
DH5⍺	F^-^ Φ80 *lacZ*ΔM15Δ (*lacZYA-argF*)U169 *deoR rec*A1 *end*A1 *hsd*R17 (r_k_^-^ m_k_^+^) *pho*A *sup*E44 λ^−^*thi*-1 *gyr*A96 *rel*A1	(76)
NiCo21 (DE3)pLysSRARE2	*can::CBD fhuA2 [Ion] ompT gal (λ DE3) [dcm] amA::CBD sly::CBD glmS6Ala* Δ*hsdS* λ *DE3* = λ *sBamHIo* Δ*EcoRI-B int::(lacI::PlacUV5::T7 gene1) i21* Δ*nin5* pLysSRARE2	New England Biolabs
Rosetta2 (DE3)pLysS	F^-^*ompT hsdS*_*B*_ (*r*_*B*_^-^*m*_*B*_^-^) *gal dcm* (DE3) pLysSRARE2 (Cam^R^)	Novagen
*Saccharomyces cerevisiae*		
RS453	*MAT***a***ade2-1 his3-11,15 leu2-3,112 trp1-1 ura3-52*	(77)
*Derivative*		
SS1026	*pah1*Δ*::TRP1*	(40)
SS1132	*pah1*Δ*::TRP1 nem1*Δ*::HIS3*	(78)
W303-1A	*MAT***a***ade2-1 can1-100 his3-11,15 leu2-3,112 trp1-1 ura3-1*	(79)
*Derivative*		
GHY57	*pah1*Δ*::URA3*	(1)
Human		
HepG2 (HB-8065)	Liver cancer cell line exhibiting epithelial-like morphology	ATCC
Plasmid		
pET-15b	*E. coli* expression vector with N-terminal His_6_-tag fusion	Novagen
*Derivative*		
pGH313	*PAH1* coding sequence insertion	(1)
pGS108	*PAH1*(ΔRP) coding sequence insertion	(80)
pET-28b(+)	*E. coli* expression vector for C-terminal His_6_-tag fusion (Kan^R^)	Novagen
*Derivative*		
pGH322	*LPIN1α* coding sequence insertion	(4)
pGH327	*LPIN1β* coding sequence insertion	(4)
pGH321	*LPIN1γ* coding sequence insertion	(4)
YEp351	High-copy number *E.coli*/yeast shuttle vector with *LEU2*	(81)
*Derivative*		
pGH311	*PAH1* coding sequence insertion	(1)
pYES2	High-copy number *E.coli*/yeast shuttle vector with *URA3* and *GAL1* promoter	Thermo Fisher Scientific
*Derivative*		
pGH452	*PAH1*-PtA coding sequence insertion with protein A tag	(82)
pGS104	*PAH1*(ΔRP) coding sequence insertion with protein A tag	(80)
pGH465	*PAH1*-CC coding sequence insertion with protein A tag	(83)

Bold “a” is the correct genetic designation.

To overexpress the phosphorylated forms of Pah1, Pah1-ΔRP, and Pah1-CC from *S. cerevisiae*, the *pah1*Δ *nem1*Δ strain (SS1132) harbored pGH452, pGS104, and pGH465 respectively (78, 80). The expression host, which lacks the Nem1-Spo7 complex, ensures the hyperphosphorylation of Pah1 (40, 82). The plasmid-bearing SS1132 cells were inoculated into SC-Ura-2% glucose at A_600__nm_ = 0.1 and incubated at 30°C for 24 h. The saturated cultures were harvested by centrifugation at 1,500 *g* for 10 min, and cell pellets were resuspended to A_600__nm_ = 0.4 in 2 l of induction media (SC-Ura-1% raffinose-2% galactose) and incubated at 30°C until A_600__nm_ = 1.0. For overexpression and purification of the unphosphorylated forms of Pah1 and Pah1-ΔRP, *E. coli* NiCo21(DE3)pLysS RARE2 harboring pGH313 and pGS108 (80), respectively, was grown to A_600__nm_ = 1.0 at 37°C in 1 l LB containing chloramphenicol (34 μg/ml) and ampicillin (100 μg/ml). For overexpression and purification of the unphosphorylated forms of lipin 1α, β, and γ, *E. coli* Rosetta2(DE3)pLysS harboring pGH322, pGH327, and pGH321 (4), respectively, was grown to A_600__nm_ = 0.5 at 37°C in 1 l of LB medium containing kanamycin (30 μg/ml) and chloramphenicol (34 μg/ml). Protein expression was induced by the addition of 1 mM isopropyl-β-D-thiogalactoside.

To examine the effect of sertraline on yeast growth, cells were incubated in RPMI-1640 (supplemented with 0.2% glucose and 165 mM MOPS, adjusted to pH 7 with NaOH) or SC-0.2% glucose media in a 24-well plate (GenClone, product number 25–102) covered with Breathe-EZ membrane (Diversified Biotech). The plate was incubated in a synergy H1 plate reader (BioTek) at 30°C with shaking, measuring A_600 nm_ every three minutes for 24 h. The effect of sertraline on the growth of WT and *pah1*Δ mutant cells in RPMI-1640 and SC-0.2% glucose media was examined in 96-well U-bottom plates (Falcon, product number 351177) using a standard Clinical and Laboratory Standards Institute broth microdilution assay (85). The cell density was determined by measurement of A_600 nm_ after a 48-h static incubation using a SpectraMax M2e microplate reader (Molecular Devices). To observe the effect of rescuing the sertraline-supplemented *pah1*Δ mutant (strain GHY57) with *PAH1*, cells overexpressing Pah1 (plasmid pGH311) or not (plasmid YEp351, vector control) were grown in SC-2% glucose to saturation before being re-inoculated at A_600 nm_ 0.1 in the same medium in the presence or absence of 200 μM sertraline. A_600 nm_ readings were taken every 2 h for 26 h. To examine the effect of sertraline on growth of cells overexpressing the Pah1-CC, the *pah1*Δ mutant (strain SS1026) expressing Pah1 (plasmid pGH452) or Pah1-CC (plasmid pGH465) were grown in SC-2% raffinose until saturation before harvesting and re-inoculating at A_600 nm_ of 0.1 in SC-2% galactose with 100 μM sertraline. The A_600 nm_ readings were taken after 4 days of incubation.

HepG2 cells were maintained at 37°C with 5% CO_2_ in Dulbecco’s Modified Eagle Medium containing 0.45% glucose, 0.058% glutamine, 0.1% sodium pyruvate, 10% fetal bovine serum, and 1% penicillin/streptomycin. The cells were seeded in triplicate to 10 cm dishes at a density of 1.1 × 10^6^ cells/plate (10 ml volume), incubated for 6 h for adherence to the plate, and then incubated with sertraline for 18 h.

### Enzyme purification

All procedures were performed at 4°C. Protein A-tagged Pah1 (82), Pah1-ΔRP (80), and Pah1-CC (83) expressed in the *S. cerevisiae pah1*Δ *nem1*Δ mutant (SS1132) were purified from the cell extracts by affinity chromatography with IgG-Sepharose followed by anion exchange chromatography with Q-Sepharose (82). His_6_-tagged Pah1 (1) and Pah1-ΔRP (86) expressed in *E. coli* NiCo21(DE3)pLysS RARE2 were purified from the cell extracts by nickel-nitrilotriacetic acid-agarose chromatography, followed by anion exchange chromatography with Q-Sepharose (1, 86). His_6_-tagged human lipin 1 isoforms expressed in *E. coli* strain Rosetta 2(DE3)pLysS were also purified from the cell extracts by nickel-nitrilotriacetic acid-agarose chromatography (4). As described previously (1, 4, 80, 86), SDS-PAGE analysis (87) indicated that the Pah1, Pah1-ΔRP, Pah1-CC, and lipin 1 preparations were highly purified. The purified enzymes were stored at −80°C.

### Preparation of Triton X-100/PA-mixed micelles

PA dissolved in chloroform was dried in vacuo for 1 h. The dried PA was suspended in Triton X-100 to prepare Triton X-100/PA-mixed micelles (88). The mole percent of PA in the Triton X-100/PA-mixed micelle was calculated using the following formula: mol %_PA_ = 100 × [PA (molar)]/([PA (molar)] + [Triton X-100 (molar)]). The total PA concentration in the Triton X-100/PA-mixed micelles was kept below 15 mol% to ensure that the structure of the PA-mixed micelles was similar to that of pure Triton X-100 micelles (89, 90).

### PAP assay

PAP activity was measured at 30°C for 20 min by following the release of water-soluble ^32^P_i_ from chloroform-soluble [^32^P]PA (5,000–10,000 cpm/nmol) (1, 88). [^32^P]PA was enzymatically synthesized from 1, 2-dioleoyl DAG and [γ-^32^P]ATP with *E. coli* DAG kinase (88). Pah1-CC PAP activity was measured by following the formation of P_i_ from unlabeled PA using the malachite green-molybdate reagent (4, 91). The reaction mixture in a total volume of 100 μl contained 50 mM Tris-HCl (pH 7.5), 1 mM MgCl_2_, 10 mM 2-mercaptoethanol, 0.2 mM PA, 2 mM Triton X-100, and 50 ng purified enzyme. The enzyme assays were conducted in triplicate; the reactions were linear with time and protein concentration. Sertraline or propranolol was dissolved in DMSO and added at the indicated amount with an equivalent volume of DMSO being added to the control reactions.

### Molecular docking

The three-dimensional structure of Pah1 predicted by AlphaFold2 (92, 93) was accessed through the ChimeraX program. The chemical structures of sertraline and propranolol were obtained from the PubChem database. The AutoDockTools graphical user interface was used for the preparation and execution of the docking simulations. AutoGrid4 was used for the generation of the atomic interaction maps (grids) used by AutoDock4 for the molecular docking simulations (94, 95). The Lamarckian genetic algorithm was used to predict 100 docked states of sertraline or propranolol to Pah1 with 25,000,000 evaluations of the docked state per run. The results were visualized in the PyMol program.

### Hydrogen–deuterium exchange mass spectrometry

The hydrogen–deuterium exchange mass spectrometry experiments followed the protocol of Zandarashvili *et al.* (96) as modified for Pah1 as follows. For deuterium exchange experiments, Pah1 at 2.3 mg/ml was incubated with 6 mM sertraline or propranolol with a 1% DMSO final concentration for at least 30 min before a 10-fold dilution into exchange buffer (25 mM HEPES, 150 mM NaCl, 5 mM TCEP, in ∼90% D_2_O) or used in the apo form for comparison. A time course was done from 20 s to 60 min at 21˚C for all conditions. Complete details of the methodology are found in supplemental Table S1.

### Lipid analysis

*S. cerevisiae* cells (5.0 × 10^6^ cells/ml) were labeled to steady state with 1 μCi/ml of [2–^14^C]acetate for 12 h at 30°C in SC-0.2% glucose with and without 163 μM sertraline. Lipids were extracted (97) from ∼ 8 × 10^7^ cells and analyzed by one-dimensional TLC with silica gel 60 plates using the solvent system containing hexane/diethyl ether/glacial acetic acid (40:10:1, v/v) (98). Radiolabeled lipids were visualized by phosphorimaging with a Storm 860 Molecular Imager (GE Healthcare) and quantified with ImageQuant software using a standard curve of [2–^14^C]acetate. The amounts of TAG and phospholipids were normalized to total lipids on TLC plates. HepG2 cells were incubated with 10 μM sertraline using DMSO as a vehicle. After 18 h of incubation, 1.5 × 10^6^ cells were collected and homogenized in chloroform/methanol (2:1, v/v), followed by the addition of acidified saline and centrifugation for phase separation (99). The organic phase was dried under nitrogen, reconstituted in chloroform/methanol (2:1, v/v), spotted onto Silica Gel 60 TLC plates, and developed in heptane/isopropyl ether/acetic acid (60:40:3, v/v). Plates were dried, dipped in the solution of 10% copper sulfate in 10% phosphoric acid, dried in air, and heated at 100°C for 40 min. Densitometry of charred bands corresponding to TAG and phospholipids was performed with ImageJ software (99) and normalized to the density of all lipids on the TLC plates.

### Lipid droplet analysis

Lipid droplets in exponential-phase cells were stained with the fluorescent dye BODIPY 493/503 (32, 100). The green fluorescence signal of the lipid droplets was observed using a Nikon Eclipse Ni-U microscope with the EGFP/FITC/Cy2/AlexaFluor 488 filter, recorded by the DS-Qi2 camera, and subjected to imaging analysis with the NIS-Elements BR software. The number of cellular lipid droplets was determined by examination of ≥ 150 cells.

### Protein quantification

Protein concentration was determined by the Bradford protein-dye binding assay (101) using bovine serum albumin as the standard.

### Data analysis

Enzyme kinetic parameters were determined using the enzyme kinetics module of the SigmaPlot software. SigmaPlot software was utilized for statistical analysis; *P* values < 0.5 were taken to be statistically significant.

## Results

### Sertraline inhibits the phosphorylated and unphosphorylated forms of Pah1 PAP

**Fig. 2:**
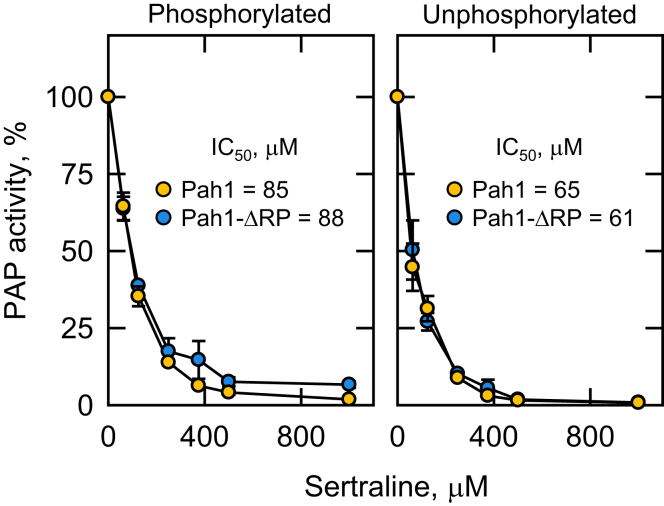
Effect of phosphorylation on the sertraline-mediated inhibition of Pah1 PAP activity. Pah1 and Pah1-ΔRP expressed in *S. cerevisiae* and *E. coli* were purified and assayed for PAP activity with varying concentrations of sertraline. The surface concentration of PA in the PAP assay was maintained at 2.4 mol %. The specific activities of Pah1 and Pah1-ΔRP from *S. cerevisiae* were 1.0 and 2.5 μmol/min/mg, respectively, and from *E. coli* 3.7 and 3.4 μmol/min/mg, respectively. The values are an average of three separate experiments ± SD (error bars). Some error bars are hidden behind the circles.

We examined the effect of sertraline on the PAP activity of Pah1 from *S. cerevisiae* by measuring the release of water-soluble ^32^P_i_ from chloroform-soluble [^32^P]PA (88). The substrate PA was delivered to the assay as a uniform Triton X-100/PA-mixed micelle to mimic the membrane surface for catalysis (102). In the first set of experiments, we utilized the phosphorylated form of the enzyme that was isolated from *S. cerevisiae* cells lacking Nem1-Spo7, a protein phosphatase complex that dephosphorylates Pah1 (40, 86, 103). PAP activity was measured at a subsaturating concentration of PA (ie, 2.44 mol %) in the Triton X-100/PA-mixed micelles (1, 102, 104) to readily observe the inhibitory effect of sertraline (105, 106, 107). Sertraline in the assay mixture caused a dose-dependent inhibition (IC_50_ = 85 μM) of Pah1 PAP activity (Fig. 2, left). It similarly inhibited (IC_50_ = 88 μM) Pah1-ΔRP (80), a Pah1 truncation variant that lacks the fungal-specific RP domain and phosphorylated less efficiently in *S. cerevisiae* cells (Fig. 2, left).

In the second set of experiments, we examined the sertraline effect on the PAP activity of unphosphorylated Pah1 and Pah1-ΔRP. The lack of enzyme phosphorylation is ensured by its heterologous expression in *E. coli*, which lacks the protein kinases that phosphorylate the enzyme (78). Again, sertraline caused a dose-dependent inhibition of unphosphorylated Pah1 (IC_50_ = 65 μM) and Pah1-ΔRP (IC_50_ = 61 μM) (Fig. 2, right). The IC_50_ values of sertraline for the inhibition of unphosphorylated Pah1 and Pah1-ΔRP were slightly lower (1.3-fold and 1.4-fold, respectively) than those for the inhibition of the phosphorylated ones. Overall, these data indicate that the phosphorylation state of Pah1 or the presence of the RP domain does not significantly affect the ability of sertraline to inhibit its PAP activity.

### Kinetics of the sertraline- and propranolol-mediated inhibitions of Pah1 PAP activity

**Fig. 3:**
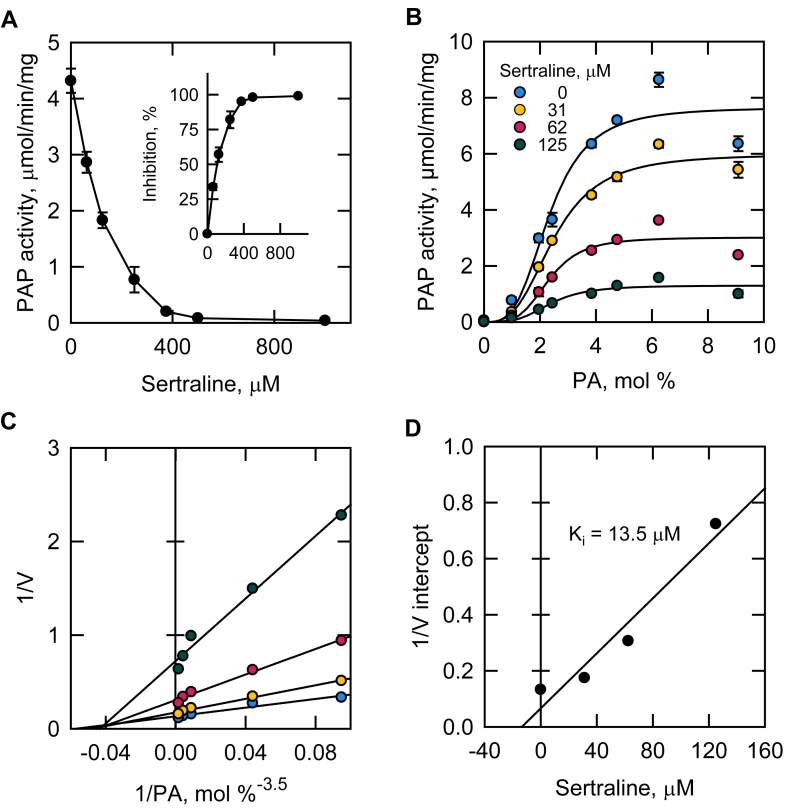
Effect of sertraline on the kinetics of Pah1 PAP activity. A: Pah1 expressed in *E. coli* was purified and assayed for PAP activity with the indicated concentrations of sertraline. The PA surface concentration was maintained at 2.4 mol %. The inset is a replot of the sertraline-mediated inhibition of PAP activity. B: Pah1 PAP activity was measured as a function of the PA surface concentration (mol %) with the indicated concentrations of sertraline. C: double reciprocal plot of the data in panel B where the PA surface concentration was raised to the average Hill number of 3.5. D: replot of the 1/V intercept values obtained from panel C versus the sertraline concentration. The values shown in A and B are an average of three separate experiments ± SD (error bars). Some error bars are hidden behind the circles.

To determine the inhibitory mechanism of sertraline, we examined the kinetics of Pah1 PAP activity (Fig. 3) with the unphosphorylated form of the enzyme, which mimics the functional (i.e., dephosphorylated) form in the cell (108, 109). The enzyme activity, which was inhibited by sertraline in a dose-dependent manner (Fig. 3A), was measured with respect to the surface concentration (mol %) of PA with varying concentrations of sertraline (Fig. 3B). As described previously (1, 12, 80), in the absence of sertraline, Pah1 PAP activity exhibits positive cooperative kinetics with the *K*_m_ value and Hill number being 2.3 mol % and 3.3, respectively. The enzyme activity was reduced by sertraline in a dose-dependent manner at each PA surface concentration. Sertraline caused a decrease in the *V*_max_ values for the PAP reaction but did not significantly affect the *K*_m_ values and Hill numbers (supplemental Table S2). Typically, double-reciprocal plots are constructed from the primary data to observe the type of inhibition exerted by an inhibitor (110). The cooperative behavior of PAP activity with respect to PA prevented the construction of these plots directly. Accordingly, the data from Fig. 3B were transformed to double reciprocal plots where the PA surface concentration was raised to an average Hill number of 3.5 (Fig. 3C) (110). Raising the PA concentration to its Hill number resulted in a family of straight lines that nearly intersected on the x-axis, a pattern typical of a non-competitive enzyme inhibition (110). A replot of the 1/V intercepts from Fig. 3C versus the sertraline concentration resulted in a straight line where the x-axis intercept is equal to the *K*_*i*_ for sertraline of 13.5 μM (Fig. 3D) (110).

**Fig. 4:**
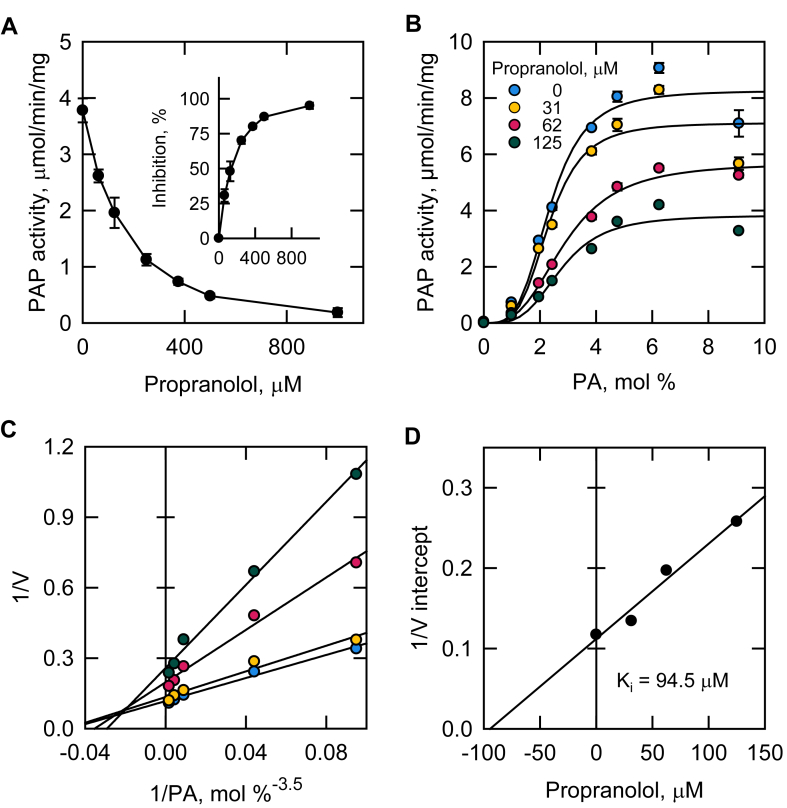
Effect of propranolol on the kinetics of Pah1 PAP activity. A: Pah1 expressed in *E. coli* was purified and assayed for PAP activity with the indicated concentrations of propranolol. The PA surface concentration was maintained at 2.4 mol %. The inset is a replot of the propranolol-mediated inhibition of PAP activity. B: Pah1 PAP activity was measured as a function of the PA surface concentration (mol %) with the indicated concentrations of propranolol. C: double reciprocal plot of the data in panel B where the PA surface concentration was raised to the average Hill number of 3.5. D: replot of the 1/V intercept values obtained from panel C versus the propranolol concentration. The values shown in A and B are an average of three separate experiments ± SD (error bars). Some error bars are hidden behind the circles.

Propranolol, a commonly used PAP inhibitor (4, 50, 51, 52, 53, 54, 55, 56, 57, 58, 59, 60), was examined for a basis of comparison to the inhibitory effects of sertraline. Propranolol inhibited PAP in a dose-dependent manner (Fig. 4A) with varying surface concentrations of PA (Fig. 4B). Like sertraline, propranolol caused a decrease in *V*_max_ values and did not majorly affect the cooperative behavior of PAP activity with respect to the surface concentration of PA (Fig. 4B and supplemental Table S2). However, relatively small changes in *K*_m_ values were observed (Fig. 4C and supplemental Table S2). The *K*_*i*_ value of 94.5 μM was determined from the replot of 1/V intercepts from Fig. 4C versus the propranolol concentration (Fig. 4D).

### The Pah1 catalytic core is the target for the sertraline- and propranolol-mediated inhibitions of PAP activity

**Fig. 5:**
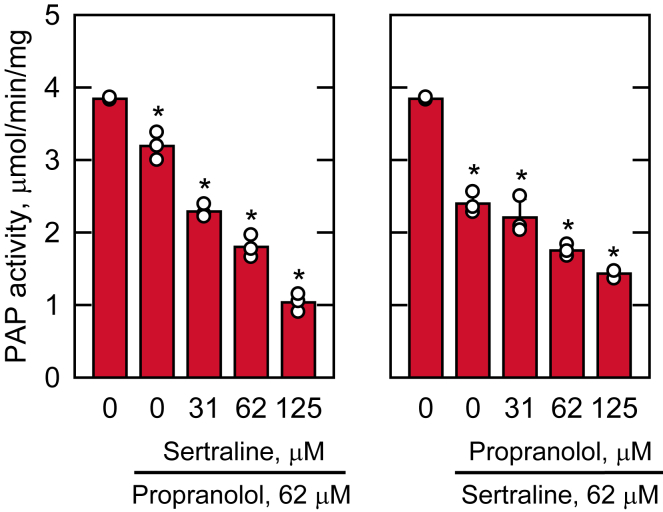
Combined effects of sertraline and propranolol on Pah1 PAP activity. Pah1 expressed in *E. coli* was purified and assayed for PAP activity with the indicated concentrations of sertraline or propranolol in the presence of 62 μM propranolol or sertraline, respectively. The PA surface concentration was maintained at 2.4 mol %. The values are an average of three separate experiments ± SD (error bars). The 0 μM inhibitor data is the same for both plots. Some error bars are hidden behind the circles. The individual data points are also shown. ∗*P* < 0.05 versus no inhibitor.

We questioned whether the inhibitory effects of PAP activity by sertraline and propranolol are additive or synergistic. To address this question, PAP activity was measured at a subsaturating surface concentration of PA in the presence of sertraline, propranolol, or both (Fig. 5). When sertraline and propranolol were fixed at the concentration of 62 μM, they exhibited a partial inhibitory effect by reducing Pah1 PAP activity by 38% (Fig. 5, right) and 17% (Fig. 5, left), respectively. As discussed above, sertraline elicited a stronger inhibitory effect when compared with propranolol. The addition of sertraline to propranolol and vice versa resulted in further inhibition of PAP activity in a dose-dependent manner (Fig. 5). At the 1:1 M ratio of sertraline and propranolol (ie, each at the concentration of 62 μM) the two drugs together inhibited Pah1 PAP activity by ∼ 50%. This result shows that sertraline exhibits an additive effect on the inhibition of Pah1 PAP when combined with propranolol, and vice versa.

We sought evidence for the interaction of sertraline or propranolol with Pah1 by hydrogen-deuterium exchange mass spectrometry. Good overall peptide coverage was obtained with approximately 90% sequence coverage, with most of the missing sequences predicted to be disordered (supplemental Table S1). Unfortunately, with a time-course of 20 s to 1 h, we failed to see protected peptides by this method, suggesting that the inhibitor-binding site does not protect backbone amide exchange or it binds in a region with low baseline exchange.

**Fig. 6:**
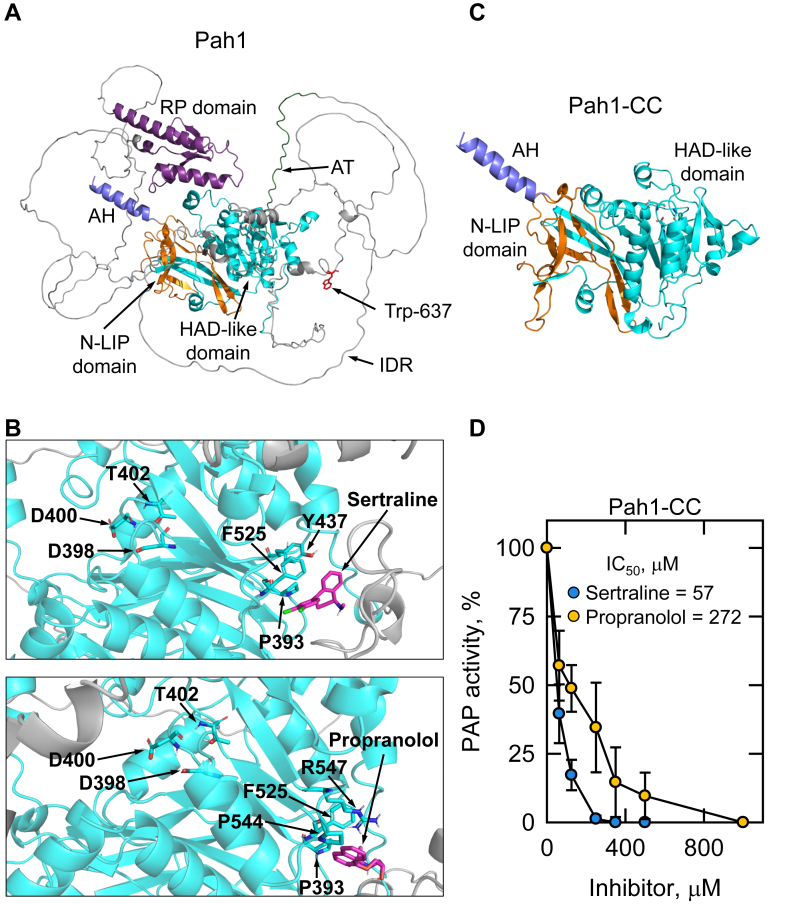
Predicted structures of *S. cerevisiae* Pah1 and Pah1-CC, molecular docking of sertraline and propranolol with Pah1, and sertraline- and propranolol-mediated inhibitions of the Pah1-CC PAP activity. A and C: the structures of Pah1 and Pah1-CC are predicted with AlphaFold and visualized using the PyMol program. The positions of the N-LIP and HAD-like catalytic domains, amphipathic helix (AH), regulation of phosphorylation (RP) domain, acidic tail (AT), intrinsically disordered regions (IDR), and Trp-637 that is contained in the WRDPLVDID domain are indicated. B: the interaction of sertraline (upper) or propranolol (lower) with Pah1 was predicted using the AutoDock4 program. The AlphaFold structure of Pah1 was used for docking simulations, which were visualized using the PyMol program. Portion of the HAD-like domain, light blue; sertraline or propranolol, pink; oxygen atoms, red; nitrogen atoms, dark blue; chlorine atoms, green. D: Pah1-CC expressed in *S. cerevisiae* was purified and assayed for PAP activity with the indicated concentrations of sertraline or propranolol. The PA surface concentration was maintained at 2.4 mol %. The specific activity of Pah1-CC was 1.3 μmol/min/mg. The values are an average of three separate experiments ± SD (error bars). Some error bars are hidden behind the circles.

Instead, we utilized the AutoDock4 (94) program with the Lamarckian genetic algorithm to predict the interaction of sertraline and propranolol with the AlphaFold (92, 93) structure of Pah1 (Fig. 6A). When sertraline and propranolol were docked to Pah1, the most frequent docked states of the inhibitors were similar, displaying interactions with the HAD-like domain that contains Asp-398, Asp-400, and Thr-402 of the D*X*D*X*(T/V) catalytic motif (Fig. 6B). Sertraline is predicted to be in close proximity to Pro-393, Tyr-437, and Phe-525 (Fig. 6B, upper) whereas propranolol is predicted to interact with Pro-393, Phe-525, Pro-544, and Arg-547 (Fig. 6B, lower), which are allosteric to the catalytic site residues. The inhibitors sertraline and propranolol interacted with the HAD-like domain in these conformations at the frequency of 42% and 15%, respectively, of the docked states (supplemental Fig. S1). The predicted *K*_*i*_ values for sertraline and propranolol derived from the molecular docking simulations were 5.9 and 87.1 μM, respectively, which are similar to the *K*_*i*_ values (13.5 and 94.5 μM, respectively) determined by the detailed kinetic analyses (Figs. 3 and 4).

Given the prediction that sertraline and propranolol interact with an allosteric site in the HAD-like domain, we tested if the inhibitors would affect the PAP activity of purified Pah1-CC (catalytic core). Pah1-CC is composed of the catalytic core (N-LIP and HAD-like domains, amphipathic helix, and the WRDPLVDID domain) and lacks all non-catalytic regulatory sequences (i.e., intrinsically disordered regions, RP domain, and acidic tail) of Pah1 (83) (Fig. 6C). Pah1-CC is enzymatically competent in vitro and in vivo, but is not regulated for its subcellular localization by phosphorylation and dephosphorylation (83). Both sertraline (IC_50_ = 57 μM) and propranolol (IC_50_ = 272 μM) inhibited the PAP activity of Pah1-CC (Fig. 6D) indicating that the catalytic core is involved with the inhibition of PAP activity.

### Sertraline and propranolol inhibit the PAP activity of human lipin 1 isoforms

**Fig. 7:**
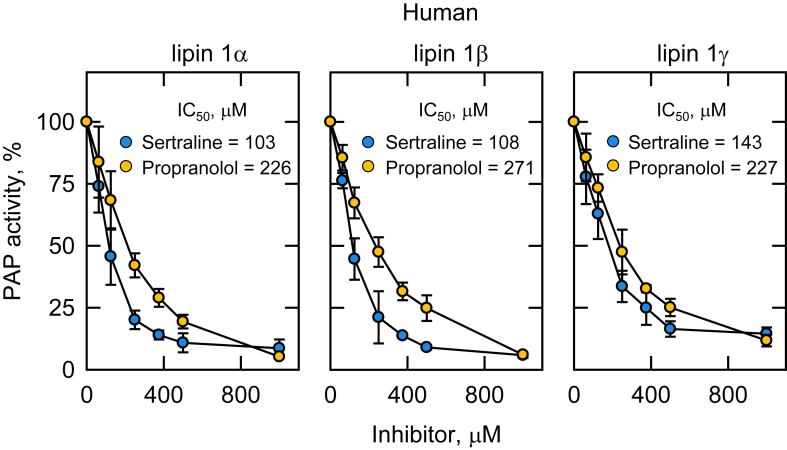
Effects of sertraline and propranolol on the PAP activity of human lipin 1 isoforms. Human lipin 1α (left), lipin 1β (middle), or lipin 1γ (right) expressed in *E. coli* was purified and assayed for PAP activity with the indicated concentrations of sertraline or propranolol. The PA surface concentration was maintained at 2.4 mol %. The specific activities of the lipin 1α, β, and γ isoforms were 1.4, 1.9, and 0.6 μmol/min/mg. The values are an average of three separate experiments ± SD (error bars). Some error bars are hidden behind the circles.

The homologous Pah1 enzyme in humans is known as lipin 1, which exists in three isoforms, namely lipin 1α, β, and γ (2, 3, 4, 111). Each of the isoforms was heterologously expressed in *E. coli*, which allowed for their isolation in the unphosphorylated state (4). The effect of sertraline on lipin 1 PAP activity was examined in the same assay condition used for *S. cerevisiae* Pah1 using the PA surface concentration of 2.4 mol % in the Triton X-100/PA-mixed micelle. Sertraline inhibited the three isoforms of lipin 1 in a dose-dependent manner (Fig. 7), and its IC_50_ values for the α, β, and γ isoforms were 103, 108, and 143 μM, respectively. Like sertraline, propranolol inhibited all three isoforms of lipin 1 in a dose-dependent manner, and its IC_50_ values for the α, β, and γ isoforms were 226, 271, and 227 μM, respectively. The IC_50_ values of sertraline for lipin 1 isoforms were ∼2-fold lower than those of propranolol, indicating that sertraline is a better PAP inhibitor.

### Sertraline-mediated growth inhibition is dependent on culture medium

**Fig. 8:**
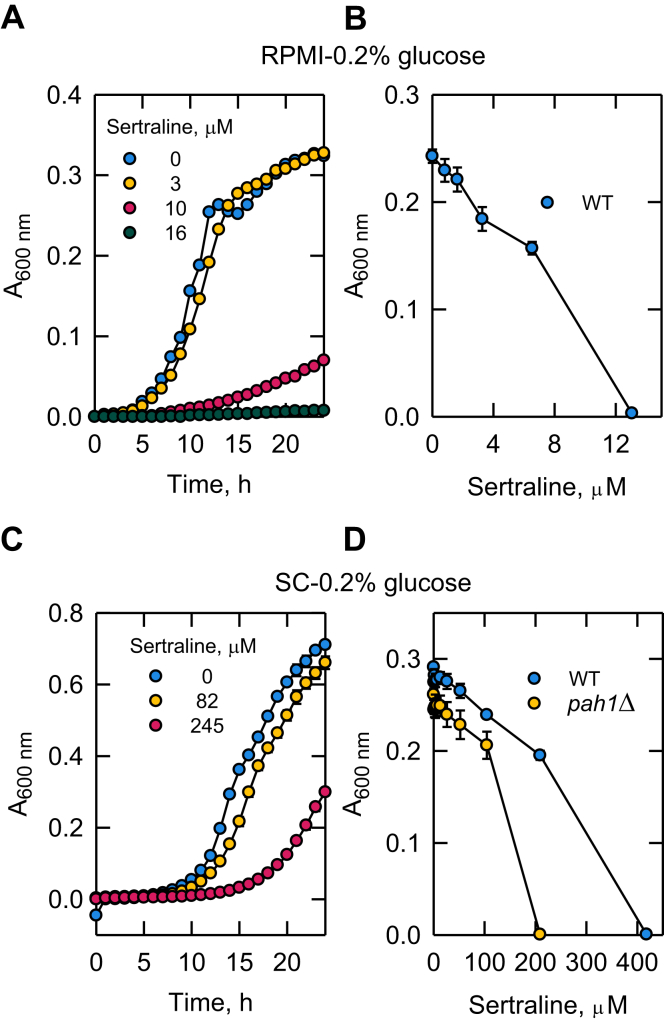
Sertraline-mediated inhibition of yeast growth is influenced by culture medium. A and C: WT *S. cerevisiae* cells (RS453) were incubated with shaking at 30°C in the indicated growth medium with varying concentrations of sertraline. The data shown is the average of two separate experiments. B and D: exponential WT (RS453) or *pah1*Δ mutant (SS1026) cells were incubated without shaking at 30°C in the indicated growth medium with varying concentrations of sertraline. The A_600 nm_ values were measured after 2 days incubation. The data are means ± SD (error bars) from three separate experiments.

The effect of sertraline on the exponential growth of *S. cerevisiae* cells was examined in two types of chemically defined synthetic medium, RPMI-0.2% glucose and SC-0.2% glucose. RPMI medium, which is limited in nutrients needed for the growth of *S. cerevisiae*, is commonly used for drug susceptibility testing of pathogenic yeast (112, 113, 114), whereas SC medium is optimized for the growth of *S. cerevisiae* (76, 84). The glucose concentration of the growth media was lowered to 0.2% to readily observe the inhibitory effect of sertraline. The drug added to the growth medium caused the inhibition of exponential growth, and its inhibitory effect was much stronger in the RPMI medium (Fig. 8A vs. C). For example, 10 μM sertraline was enough in the RPMI-0.2% glucose medium to greatly reduce cell growth, whereas 245 μM of the drug was required in SC-0.2% glucose medium to elicit a similar reduction of cell growth. In another experiment, exponential phase cells were statically incubated for 2 days with different concentrations of sertraline. In this case, the drug inhibited cell growth in a dose-dependent manner (Fig. 8B, D). The concentration of sertraline that abolishes cell growth was ∼30-fold less in RPMI-0.2% glucose medium when compared with that in SC-0.2% glucose medium.

### High-copy expression of *PAH1* rescues the sertraline-mediated reductions in growth and lipid droplet formation in *pah1*Δ mutant cells

The *pah1*Δ mutant, which lacks the PAP activity of Pah1 (1), exhibits a myriad of deleterious phenotypes that are caused by a defect in balancing cellular levels of PA and DAG (1, 15, 41). Accordingly, we questioned what effect sertraline has on the growth of *pah1*Δ cells. For this experiment, we cultured the mutant in SC-0.2% glucose medium because of its growth defect in RPMI-0.2% glucose. The *pah1*Δ mutant was more sensitive to sertraline than the WT control, and its growth was completely inhibited at the drug concentration of 200 μM (Fig. 8D).

**Fig. 9:**
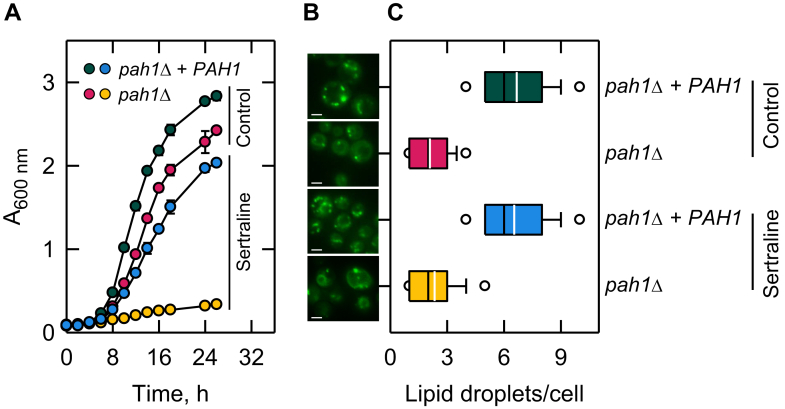
High-copy expression of *PAH1* rescues sertraline-mediated reductions in growth and lipid droplet formation in *pah1*Δ mutant cells. A: *pah1*Δ cells (strain GHY57) with (plasmid pGH311) and without (plasmid YEp351, vector control) high-copy expression of *PAH1* were grown in SC-2% glucose in the absence and presence of 200 μM sertraline where indicated. The data are means ± SD (error bars) from three separate experiments. B: cells after 14 h of growth were stained with BODIPY 493/503 to visualize cellular lipid droplets by fluorescence microscopy. White bar, 10 μm. C: lipid droplets numbers were quantified from ≥150 cells (≥4 fields of view). The black and white lines are the median and mean values, respectively, and the white circle*s* are the outlier data points of the 5^th^ and 95^th^ percentile.

We questioned whether the sertraline-mediated growth inhibition of the *pah1*Δ mutant is rescued by high-copy expression of *PAH1*. We reasoned that an overexpressed level of PAP activity would more readily rescue the inhibitory effect of the drug on the mutant growth. In this experiment, the glucose concentration was raised to 2% in the SC medium to allow better growth of the *pah1*Δ mutant in the presence of sertraline. The *pah1*Δ mutant incubated in SC-2% glucose with 200 μM sertraline did grow but exhibited a severe reduction in growth (Fig. 9A). The growth defect of *pah1*Δ cells caused by the drug was rescued by the overexpression of *PAH1*. In the absence of sertraline, *pah1*Δ cells expressing *PAH1* also showed better growth when compared with those lacking the gene. However, the positive effect of *PAH1* on the growth of *pah1*Δ cells was much greater in the presence of sertraline (Fig. 9A).

As described previously (30, 73), lipid droplet formation was disrupted in *pah1*Δ cells when they were cultured in the absence of sertraline; the low lipid droplet numbers were also observed in the mutant cells treated with the drug (Fig. 9B, C). Consistent with the effect that *PAH1* gene overexpression had on the growth of sertraline-treated *pah1*Δ mutant cells, the PAP-encoding gene overexpression rescued the defect of the mutant cells in lipid droplet formation (Fig. 9B, C).

### Sertraline rescues the lethal effect of Pah1-CC overexpression on cell growth

**Fig. 10:**
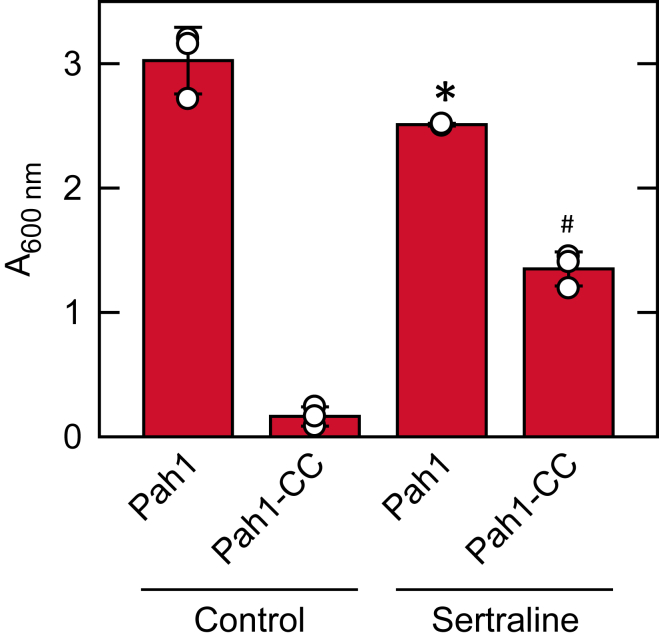
Sertraline rescues the lethal effect of Pah1-CC overexpression on cell growth. *pah1*Δ cells (SS1026) overexpressing Pah1 (pGH452) or Pah1-CC mutant variant (pGH465) were grown in SC-2% raffinose until saturation before harvesting and re-inoculating at A_600 nm_ of 0.1 in SC-2% galactose with 100 μM sertraline. The A_600 nm_ readings were taken after 4 days. The data are means ± SD (error bars) from three separate experiments. The individual data points are also shown. Some error bars are hidden behind the circles. ∗*P* < 0.05 versus Pah1 of control. #*P* < 0.05 versus Pah1-CC of control.

The excess PAP activity imparted by overexpression of the unregulated Pah1-CC variant disturbs lipid metabolism to the point of causing cell death (83). Given that sertraline inhibits the PAP activity of Pah1-CC in vitro (Fig. 6D), we reasoned that the drug might ameliorate the lethal effect of the variant in vivo. To examine this hypothesis, Pah1-CC, as well as Pah1, were overexpressed in cells incubated in the absence and presence of 100 μM sertraline; growth was measured after 4 days of incubation (Fig. 10). As expected, the overexpression of Pah1-CC prevented the growth of cells incubated without sertraline, and sertraline caused a mild inhibition of the growth of the cells expressing Pah1. The addition of sertraline to the growth medium of the cells overexpressing Pah1-CC partially rescued the inhibitory effect of the variant (Fig. 10). These results indicated that the catalytic core of Pah1 is a target for sertraline in vivo.

### Sertraline reduces TAG content in *S. cerevisiae* and human HepG2 cells

**Fig. 11:**
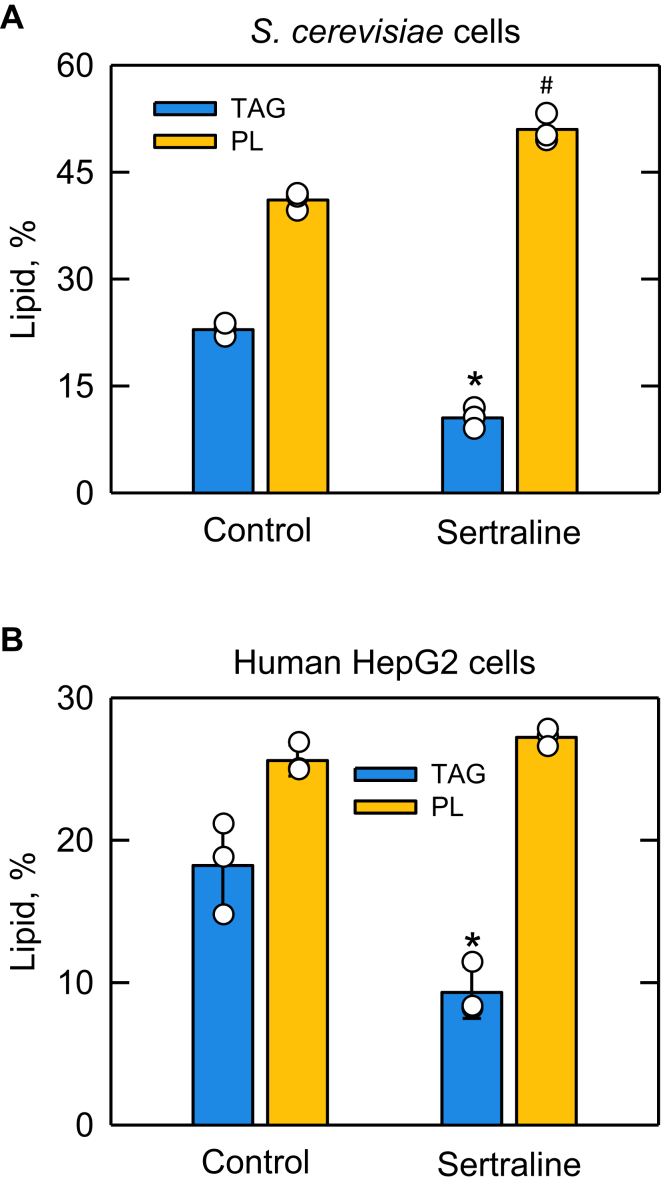
Effect of sertraline on TAG content in *S. cerevisiae* and human HepG2 cells. A: WT *S. cerevisiae* cells (RS453) were grown at 30°C for 12 h in SC-0.2% glucose medium in the absence or presence of 163 μM sertraline and [2–^14^C]acetate (1 μCi/ml). Lipids were extracted from ∼ 8 × 10^7^ cells, separated by one-dimensional TLC, and visualized by phosphorimaging analysis with ImageQuant software. The percentage shown for TAG and phospholipids were normalized to the total ^14^C-labeled lipids on the TLC plates. B: HepG2 cells in supplemented Dulbecco’s Modified Eagle Medium were allowed to adhere to individual culture plates for 6 h. The adhered cells were then incubated with 10 μM sertraline for 18 h in the same growth medium. The growth of cells during the incubation period did not differ from that of the control treated with the DMSO vehicle. Lipids were extracted from 1.5 × 10^6^ cells, separated by one-dimensional TLC, visualized by charring plates at 100°C for 40 min, and analyzed with ImageJ software. The percentage shown for TAG and phospholipids were normalized to the total charred lipid on the TLC plate. The data are means ± SD (error bars) from three separate experiments. The individual data points are also shown. Some error bars are hidden behind the circles. ∗*P* < 0.05 versus TAG of control. #*P* < 0.05 versus phospholipid of control. PL, phospholipid.

In the de novo pathway of lipid synthesis in *S. cerevisiae*, the DAG produced by Pah1 PAP activity is channeled into TAG (1, 15, 115). The DAG produced by the enzyme reaction is also utilized by mutants defective in the synthesis of the membrane phospholipids phosphatidylcholine and/or phosphatidylethanolamine if they are supplemented with choline and/or ethanolamine via the CDP-choline and/or CDP-ethanolamine branches of the Kennedy pathway (15, 116, 117). The PAP substrate PA is normally utilized for the synthesis of phosphatidylcholine and phosphatidylethanolamine, along with all other membranes phospholipids (e.g., phosphatidylinositol, phosphatidylserine, phosphatidylglycerol, cardiolipin), via the CDP-DAG pathway (15, 116, 117). Accordingly, we questioned whether the sertraline-mediated inhibition of PAP activity in vitro correlates with a change in the lipid contents in vivo. For this experiment, WT cells were grown in the absence or presence of a sublethal dose of sertraline in SC-0.2% glucose medium containing [2–^14^C]acetate. The radiolabeling was performed in the SC medium owing to the difficulty of [2–^14^C]acetate incorporation into cells grown in the RPMI medium. Following a 12 h radiolabeling, lipids were extracted, subjected to TLC analysis, and the levels of TAG and phospholipids were determined by phosphorimaging and ImageQuant analyses (Fig. 11A). In the control cells grown without sertraline, the relative amounts of TAG and phospholipids were ∼20 and ∼40%, respectively. In contrast, the TAG and phospholipid contents in the sertraline-treated cells were decreased and increased, respectively, by 2.2- and 1.2-fold (Fig. 11A). Thus, the inhibition of the PAP reaction resulted in the utilization of PA for the synthesis of phospholipids at the expense of TAG.

As in *S. cerevisiae*, the DAG produced by the PAP reaction in mammalian cells is utilized for the synthesis of TAG (44, 118). In contrast to *S. cerevisiae*, phosphatidylcholine and phosphatidylethanolamine are predominately synthesized via the Kennedy pathway from the DAG produced in the PAP reaction (44, 118). As in *S. cerevisiae*, PA is utilized, via CDP-DAG, for the synthesis of other membrane phospholipids that include phosphatidylinositol, phosphatidylglycerol, and cardiolipin (44, 118). The sertraline-mediated inhibition of human lipin 1 PAP activity prompted the analysis of lipid content in human HepG2 cells in response to the drug. In this experiment, the cells adhered to culture dishes for 6 h and were incubated with 10 μM sertraline for 18 h in supplemented Dulbecco’s Modified Eagle Medium. Following the sertraline treatment, lipids were extracted, separated by TLC, and visualized by charring. The quantification of lipid contents showed that the major effect of sertraline on HepG2 cells was shown by a 2-fold reduction in the TAG level (Fig. 11B). In contrast to the sertraline-mediated increase in phospholipids observed in *S. cerevisiae* (Fig. 11A), the relative amount of phospholipids in the HepG2 cells was not significantly affected by the drug treatment (Fig. 11B). This result is not easily explained given that phospholipids in mammalian cells are synthesized from both PA and DAG. How the sertraline-mediated inhibition of PAP activity affected their synthesis is unclear.

## Discussion

In the yeast *S. cerevisiae*, as well as in higher eukaryotic organisms, PAP plays a key role in regulating the PA/DAG balance and control of lipid synthesis (15, 17, 41, 44, 108, 119, 120). Drugs that inhibit PAP activity are useful in that they may regulate cellular functions associated with the substrate PA and product DAG (e.g., the synthesis of membrane phospholipids and the neutral lipid TAG) that ultimately control cell metabolism and growth (15, 17, 41, 44, 108, 119, 120). Of the drugs that have been shown to inhibit PAP activity (e.g., propranolol, phenylglyoxal, chlorpromazine, and bromophenol lactone) (50, 51, 52, 121, 122, 123, 124), propranolol has received the most attention. For example, propranolol has been useful in showing how PAP influences cellular physiology and disease states in mammalian cells (28, 59, 60, 61, 62, 63) and how the enzyme affects virulence and inhibits the growth of pathogenic yeast (55). Here we showed that the antidepressant drug sertraline (64) is a novel PAP inhibitor that rivals the potency of propranolol.

Using purified Pah1 from *S. cerevisiae* as a model, we showed that sertraline inhibits PAP activity by a noncompetitive mechanism affecting the *V*_max_ of the reaction with a *K*_*i*_ value (13.5 μM) in the low micromolar range. The sertraline-mediated inhibition of Pah1 PAP activity was not significantly affected by its phosphorylation, which regulates the enzyme activity (109), or by the fungal-specific RP domain required for efficient phosphorylation (80). These data also imply that sertraline does not inhibit Pah1 PAP activity by interacting with the RP domain or the intrinsically disordered regions of Pah1 leaving the structured catalytic core as the most likely target. Based on *K*_*i*_ values, sertraline was a 7-fold more effective inhibitor when compared to propranolol. Although a kinetic comparison was not performed here, we showed that sertraline inhibited the PAP activities of purified human lipin 1 isoforms and that it was a 2-fold better inhibitor based on the IC_50_ values when compared with propranolol. The IC_50_ values of sertraline for the inhibition of lipin 1α, β, and γ were similar, suggesting that the drug does not interact with the isoform-specific sequences.

In the inhibition of *S. cerevisiae* Pah1 PAP, the effects of sertraline and propranolol were additive at subsaturating concentrations of the inhibitors. Where these data cannot distinguish whether the drugs are additive because of the use of subsaturating concentrations or that the drugs target different sites on Pah1, they are consistent with sertraline being a better inhibitor than propranolol. If sertraline has a higher affinity for a preferred or shared site though, sertraline should outcompete propranolol when sertraline is added in greater amounts to the reactions which is consistent with the greater extent of inhibition seen with increasing sertraline concentrations. Molecular docking of the drugs indicated that they preferentially interact with a similar region within the HAD-like catalytic domain of the enzyme. The predicted residues of interaction are allosteric to those of the D*X*D*X*(T/V) catalytic residues. Moreover, the lack of major effects of sertraline and propranolol on the *K*_m_ value for PA is consistent with the drugs interacting with an allosteric site. That sertraline and propranolol target the HAD-like domain of Pah1 is supported by the experimental evidence that both drugs inhibited the PAP activity of the purified Pah1-CC variant that is comprised only of the catalytic core of Pah1. In a previous docking simulation study (55), propranolol was shown to interact with HAD-like domain residues of the *M. oryzae* Pah1, although not the homologous residues identified here. Whether sertraline inhibits the PAP activity and docks to the HAD-like domain of the *M. oryzae* Pah1 homolog is unknown. Taken together, these data imply sertraline and propranolol share a common preferred site of interaction with Pah1, but do not preclude multiple sites of interaction.

Prior work has shown that sertraline inhibits the growth of *S. cerevisiae* (71) as well as that of pathogenic yeasts and filamentous fungi (65, 66, 67, 68, 69, 70). Here, we showed that the inhibitory effect of sertraline on growth was dependent on the medium in which the cells were grown. RPMI, a growth medium routinely used for growing mammalian cells in culture and commonly used to assess the effectiveness of antimycotic agents in an environment found in the human body during infection (112, 113, 114), afforded greater sertraline-mediated sensitivity when compared with SC medium formulated for *S. cerevisiae* growth (76, 84). We found that the RPMI medium compromised [2–^14^C]acetate uptake and labeling of lipids in WT cells, as well as the growth of *pah1*Δ mutant cells. Accordingly, SC-0.2% glucose medium was used for these studies. In the SC-0.2% glucose medium, the *pah1*Δ mutant was much more sensitive to sertraline when compared with that of WT cells. It is well known that the *pah1*Δ mutant is already sensitive to several growth stressors (e.g., temperature, killer toxin, hydrogen peroxide, fatty acids) (1, 12, 15, 73, 125). Consequently, *S. cerevisiae*, and perhaps opportunistic fungal pathogens, would be more readily susceptible to other antimycotic agents if PAP activity is inhibited by sertraline. That Pah1 is a physiological target of sertraline is supported by the observations that the overexpression of *PAH1* in *pah1*Δ mutant cells rescued the sertraline-mediated growth inhibition of the mutant, the lethal effect of Pah1-CC overexpression in *S. cerevisiae* is rescued by sertraline supplementation, and that a sublethal dose of the drug resulted in 2-fold decrease in the TAG content. The decrease in TAG content of sertraline-supplemented HepG2 cells supports the notion that lipin 1 PAP might be a target for the drug in humans.

Whether sertraline might be used to effect remediation of lipid-based disease based on PAP inhibition in humans is unknown. In theory, the sertraline-mediated inhibition of TAG synthesis could minimize latent *Mycobacterium tuberculosis* infection that relies on host TAG for survival (126). Other potential applications include the alleviation of intestinal inflammation-driven colon cancer development (127) and suppression of SARS-CoV-2 replication (128), conditions affected by loss of lipin 1 PAP activity. Although these applications are speculative, sertraline may be used as a lead compound. Structural analogs could be used to identify the functional groups responsible for the selective serotonin reuptake inhibitor-related properties of sertraline versus the functional groups relevant to its ability to inhibit PAP activity. Structural analogs could also be used to enhance the specificity or potency of the drug as a PAP inhibitor.

Repurposing existing therapeutics is one option for developing new antimycotic strategies (65, 129, 130), and sertraline appears to be a good candidate for this purpose. Utilizing sertraline in combination therapies with preexisting antimycotics may amplify the effects of those drugs prolonging their usefulness as antifungal resistance continues to evolve (131, 132). While the data support the conclusion that the inhibitory effect of sertraline on *S. cerevisiae* growth stems from the inhibition of Pah1 PAP activity, other mechanisms are possible. The inhibitory effect of sertraline on the growth of *pah1*Δ mutant cells indicates additional targets of the drug. For example, sertraline has been shown to intercalate into phospholipid bilayers to alter membrane organization and modulate phospholipase activities in *S. cerevisiae* (71). These are additional mechanisms that would facilitate the effectiveness of the drug to inhibit cell growth.

## Data availability

All data are contained within the article or two supplemental tables and one supplemental figure.

## Supplemental data

This article contains three supplemental files (96, 133).

## Conflict of interest

The authors declare that they have no conflicts of interest with the contents of this article.
