# Supplementary material for: The antidepressant drug sertraline is a novel inhibitor of yeast Pah1 and human lipin 1 phosphatidic acid phosphatases
Source: J Lipid Res. 2024 Nov 20;66(1):100711. doi: 10.1016/j.jlr.2024.100711 (PMC11721541; doi:10.1016/j.jlr.2024.100711)

Figure S1. Histogram clustering of molecular docking conformations. The docked states of sertraline or propranolol to the AlphaFold2 structure of Pah1 were predicted 100 times using the AutoDock4 algorithm. The conformation of the docked inhibitor underwent 25,000,000 evaluations per run. Runs which predicted the same conformation of sertraline (*left*) or propranolol (*right*) interacting with Pah1 were clustered to determine the frequency of every docked state. The most frequently predicted docked states were visualized using the PyMol program and are presented in Fig. 6B.

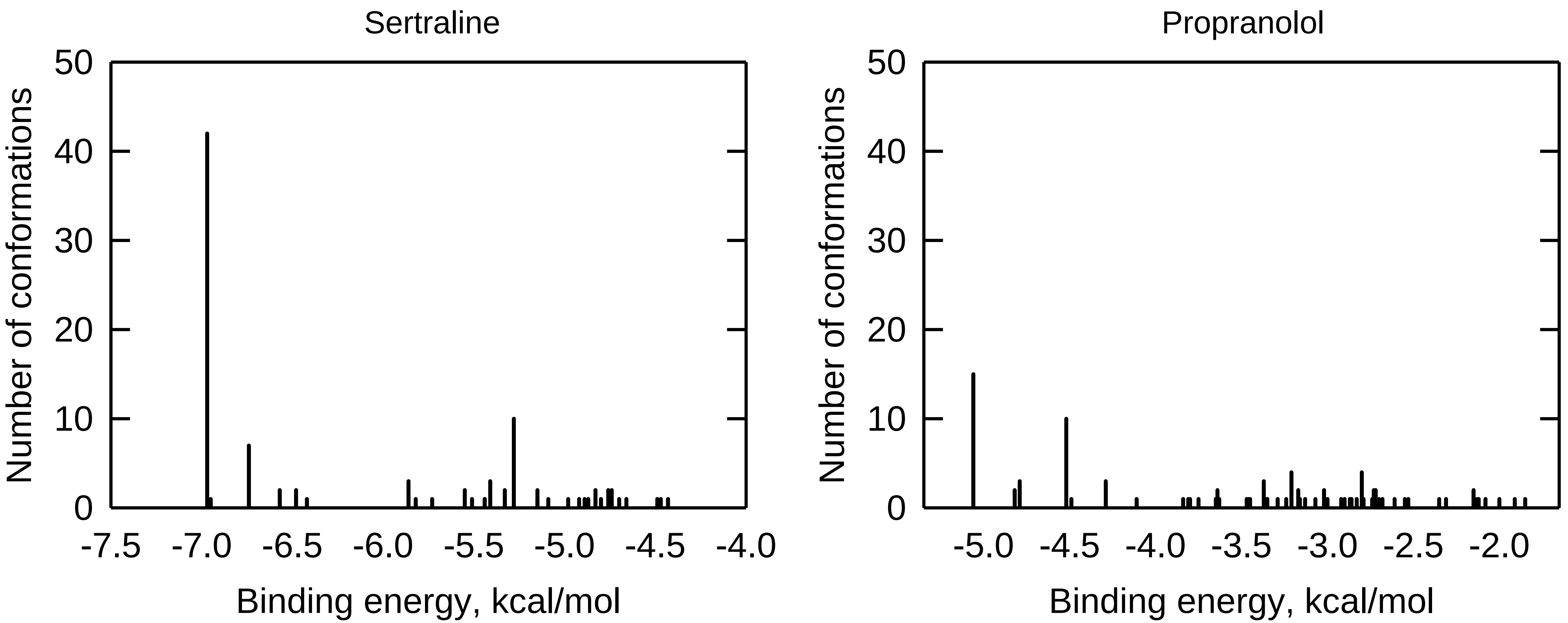

Supplement: Fig. S1 [file mmc1.pdf]
