# Supplementary material for: The antidepressant drug sertraline is a novel inhibitor of yeast Pah1 and human lipin 1 phosphatidic acid phosphatases
Source: J Lipid Res. 2024 Nov 20;66(1):100711. doi: 10.1016/j.jlr.2024.100711 (PMC11721541; doi:10.1016/j.jlr.2024.100711)
Supplement: Table S2 [file mmc3.pdf]

Table S2

Kinetic constants for Pah1 PAP activity inhibited by sertraline and propranolol. Data were calculated from the plots shown in Figs. 3B and 4B.

Inhibitor	V_{max}	K_m	Hill no.
	μmol/min/mg	*mol %*	*n*
Sertraline, μM			
0	7.6	2.3	3.3
31.25	6.0	2.5	3.1
62.5	3.0	2.3	4.0
125	1.3	2.4	3.7
Propranolol, μM			
0	8.3	2.4	3.7
31.25	7.1	2.3	3.9
62.5	5.7	2.9	3.0
125	3.8	2.8	3.6
